# *In Vitro* Effects of Budesonide on Eosinophil-Basophil Lineage Commitment

**DOI:** 10.2174/1874306400802010060

**Published:** 2008-06-13

**Authors:** Michael M Cyr, Adrian J Baatjes, Sandra C Dorman, Lynn Crawford, Roma Sehmi, Ronan Foley, Rafeul Alam, Paul O’ Byrne, Judah A Denburg

**Affiliations:** 1 Division of Clinical Immunology and Allergy, McMaster University, Hamilton, ON, Canada; 2 Division of Allergy & Immunology, National Jewish Medical & Research Center, Denver, CO, USA

**Keywords:** Budesonide, eosinophil/basophil progenitors, corticosteroids, GATA-1, IL-5.

## Abstract

IL-5 is the primary cytokine that stimulates the production and survival of eosinophils and basophils from progenitor cells. The inhaled glucocorticoid, budesonide, has been shown to exert a therapeutic effect *via *suppression of eosinophil/basophil progenitors *in vivo*. Since various steroids have exhibited the ability to enhance eosinophil/basophil progenitor differentiation, we examined the effects of budesonide *in vitro*. Bone marrow and cord blood samples were obtained and cultured in the presence of IL-5 alone or IL-5 plus budesonide. Eosinophil/basophil colony-forming units were enumerated from cultured nonadherent mononuclear cells and from purified CD34^+^ cells. CD34^+^ cells with and without budesonide were also examined for up-regulation of ERK1/2, MAPK and GATA-1 using real time-PCR. Results: i) up-regulation of eosinophil/basophil colony-forming units is due to the direct effects of budesonide on IL-5-stimulated progenitors; ii) GATA-1 is likely involved in the early amplification of eosinophil/basophil progenitor commitment leading to increased differentiation. A potential transcriptional pathway has been identified which may mediate the effects of budesonide on eosinophil/basophil lineage commitment.

## INTRODUCTION

An increase in tissue eosinophils is one of the most characteristic features of allergic inflammation. Eosinophils share a common progenitor with basophils, identified functionally as eosinophil/basophil colony-forming units (Eo/B CFU), which grow in semi-solid media in the presence of cytokines including IL-3, IL-5, and GM-CSF [1,2]. Of these cytokines, IL-5 plays a particularly important role in the eosinophil/basophil lineage-commitment of progenitor cells [3].

Our group and others have illustrated the involvement of Eo/B progenitors in allergic inflammatory disease in the following studies: i) increases in circulating Eo/B CFU in subjects with atopy and allergic rhinitis [1,4]; ii) increases in circulating Eo/B CFU during seasonal exposure to allergens [5,6]; iii) increases in circulating and bone marrow progenitors and upregulation of IL-5Rα expression after allergen inhalation challenge [7-10] and iv) increases in airway CD34^+^ cells expressing the IL-5Rα after allergen inhalation [11].

Corticosteroids are currently the most effective therapy in the treatment of asthma, acting at least in part through the suppression of Eo/B progenitors *in vivo* [9,12,13]. Inhaled steroids have been shown to decrease circulating Eo/B CFU [14] and to attenuate increases in circulating Eo/B CFU [13]in subjects with asthma. Moreover, the controlled, step-wise withdrawal of inhaled corticosteroids in atopic asthmatics leads to a rapid and sustained increase in peripheral blood Eo/B CFU, coinciding with the development of asthmatic symptoms and with peak-flow changes; the re-introduction of inhaled corticosteroids, conversely leads to resolution of symptoms, accompanied by a decrease in circulating eosinophils, basophils and Eo/B CFU [12]. In an allergen-challenge model studying subjects with asthma, a short course of inhaled corticosteroids leads to a decrease in the baseline number of bone marrow CD34^+^IL-5Rα^+ ^eosinophil progenitors and IL-5-responsive Eo/B CFU; interestingly however, inhaled corticosteroids do not prevent allergen-induced increases of either [9]. Thus, while topical corticosteroids exert suppressive effects on the differentiation of eosinophil progenitors [9,12,13], this is probably *via *the regulation of tissue cytokine expression [15], a mode of suppression insufficient to fully abrogate the marrow contribution to eosinophil recruitment* in vivo*.

Indeed, stimulatory effects of steroids on eosinophil growth and differentiation have been observed since the 1950s *in vitro *[16]. Studies of bone marrow cultures indicate that at physiological and pharmacological concentrations, hydrocortisone is able to stimulate eosinophilopoiesis [17]. More recently, others have shown, using a murine model, that glucocorticoids *enhance *bone marrow eosinophilopoiesis in normal and allergic mice [18]; the latter group also demonstrated that pre-treatment of these mice with dexamethasone for 24 hours *in vivo* resulted in *increased* progenitor responses to both GM-CSF and IL-5 *ex vivo*. These studies indicate a direct, stimulatory effect of corticosteroids on eosinophil progenitors.

Corticosteroids have revolutionized the treatment of asthma and their anti-inflammatory effects are exerted through multiple pathways. Perhaps *because* of their significant effectiveness, researchers have only recently begun examining the potential *limitations* of corticosteroids in the treatment of asthma. The ability of corticosteroids to prevent airway remodeling is conflicting [19] and recently it has been demonstrated in a murine model of asthma, that corticosteroids are not only unable to prevent mucosal sensitization but may indeed enhance the allergic response upon allergen rechallenge [20].

In this study, we examine the *in vitro* effects of a clinically active, topical corticosteroid, budesonide (BUD), on Eo/B CFU formation. We show evidence for enhancement of IL-5-mediated eosinophil growth and differentiation, and suggest an effect on the Eo/B lineage-specific transcription factor, GATA-1, as a possible explanation for this enhancement.

## MATERIALS AND METHODS

This study was approved by the Hamilton Health Sciences/McMaster University Faculty of Health Sciences Research Ethics Board, and subjects provided written informed consent to participate.

### Collection and Processing of Bone Marrow

Bone marrow aspirates (2-3 ml) were collected by a hematologist, from the iliac crest of human subjects using a bone marrow aspiration needle (16 x 2 inches; Sherwood Medical, St. Louis, Mo), into a 10ml syringe containing 1ml of sterile heparin (1000 Units/ml; Sigma Chemical Co., St. Louis, MO). Bone marrow was then transferred to a 50ml Falcon tube and diluted to 50ml with McCoy’s 5A (Gibco, New York, USA).

### Collection and Processing Cord Blood Cells

Cord blood was collected in a 60ml syringe containing 2ml of sterile heparin (1000 Units/ml; Sigma-Aldrich, Oakville, Canada). Red blood cells were sedimented with 1% Dextran (Sigma-Aldrich) at 37°C for 30 minutes and white cells were removed and diluted 1:1 with McCoy’s 5A (Gibco).

### Non-Adherent Mononuclear Cell Isolation

White cells were layered over Accuprep (Accurate Chemical and Scientific Corp., New York, USA) and subjected to density gradient separation (2000rpm for 20 minutes at room temperature). Mononuclear cells were removed and washed (1500 rpm for 10 minutes at room temperature) in McCoy’s 5A (Gibco). After washing, cells were resuspended in McCoy’s 5A supplemented with 15% FBS (Sigma-Aldrich), 1% Penicillin-Streptomycin (Gibco) and 1% 2-ME, and incubated at 37°C and 5% CO_2_ in a plastic flask (Corning Inc., New York, USA) for 2 hours at 1x10^6^ mononuclear cells/ml. The remaining non-adherent mononuclear cells (NAMNC) were collected and washed.

### CD34^+^ Progenitor Cell Isolation

CD34^+^ progenitor cells were isolated from NAMNC using a CD34 progenitor cell isolation kit (Miltenyi Biotec, Auburn, CA) *via *indirect labeling and positive selection techniques [21]. NAMNC were washed once in cold magnetic activated cell sorting (MACS) buffer and then resuspended in cold MACS buffer. NAMNC were incubated with a blocking antibody and primary anti-CD34 (QBEND/10 epitope) hapten-conjugated antibody for 15 minutes at 4^°^C. Cells were then washed once and resuspended in cold MACS buffer. NAMNC were then magnetically labeled *via *incubation with a secondary anti-hapten Microbead conjugated antibody. Labeled cells were then washed, re-suspended and then run through a MS column using the MiniMACS system (Miltenyi Biotec). The positive fraction was eluted in McCoy’s 3^+^. The total number of CD34^+^ cells was determined by performing a cell count using a hemocytometer (Neubauer Chamber, Hausser Scientific).

### Methylcellulose Colony Assays

Methylcellulose colony assays for bone marrow and cord blood (CB) Eo/B CFU were performed as previously described using peripheral blood [4]. Bone marrow NAMNC were cultured at 2.5 x 10^5^ cells per 35 x 10-mm tissue culture dish (Falcon Plastics, Oxnard, CA) with IL-5 (10ng/ml) (Pharmingen, Markham, ON, Canada) ± BUD (10^-8^-10^-7^) (AstraZeneca, Lund, Sweden). Cells were cultured in 0.9% methylcellulose (Sigma) with Iscove’s modified Dulbecco’s medium (Gibco) and 20% fetal bovine serum supplemented with 1% penicillin-streptomycin and 5 x 10^-5^ M 2-mercaptoethanol. CB CD34^+^ cells were cultured at 8 x 10^3^ per 35 x 10-mm tissue culture dish in the presence of IL-5 (1 ng/ml) ± BUD (10^-9^-10^-7^).

Cultures were incubated for 14 days at 37 ^°^C and 5% CO_2_. Day 14 Eo/B type granulocyte colonies (>40 cells) were enumerated in two replicate methylcellulose plates under inverted light microscopy and expressed as Eo/B CFU per cells plated. The Eo/B colonies were identified by morphology as tight, granulated, round, refractile cell aggregations [1,22].

### Flow Cytometric Specific Mean Fluorescence Intensity (sMFI) Assay of CB CD34^+^ Cells

Isolated CD34^+^ cells were stimulated with IL-5 (1 ng/ml) alone, or IL-5 (1 ng/ml) with BUD (10^-8^M), and incubated in RPMI complete (10^6^ cells/ml; Gibco) in plastic flasks for 24 hours at 37^°^C and 5% CO_2_. After stimulation, CB CD34^+^ cells were stained and analyzed by flow cytometry as previously described [10]. Briefly, cells were washed with cold 0.1% PBS/NaN_3_ solution (PBS containing 0.1% NaN_3 _and 0.5% BSA; Sigma). A minimum 50 x 10^3^ CD34^+^ cells per tube were stained with saturating amounts of biotinylated anti-IL-5Rα (Roche Laboratories, Ghent, Belgium) or biotinylated IgG_1_ isotype control antibodies (Becton Dickinson, Mississauga, Ontario, Canada) for 30 minutes at 4^°^C. The cells were then washed and stained with streptavidin-conjugated Cychrome (Becton Dickinson), along with saturating concentrations of anti-CD45 FITC (Becton Dickinson) and anti-CD34-PE (Becton Dickinson) in a final volume of 100 μl of 0.1% PBS/NaN_3_ solution for 30 minutes at 4^°^C. Cells were then washed and fixed in 500 μl of PBS containing 1% paraformaldehyde (Sigma). Stained cells were stored in the dark at 4^°^C until ready for analysis. Stained cells were analyzed using a FACScan flow cytometer (Becton Dickinson Instrument Systems, BDIS, Mississauga, Canada). Each measurement contained 30,000 events and subsequent analysis of the acquired data was performed using the PC lysis software (BDIS). The sMFI of the IL-5Rα was obtained by subtracting the MFI of the isotype control from the MFI for the IL-5Rα for both stimulated and unstimulated samples.

### Signal Molecule Activation Assay

Stimulated and un-stimulated bone marrow CD34^+ ^cells were lysed in RIPA buffer (50 mM Tris-HCl (pH 7.4), 150 mM NaCl, 1 mM EGTA, 0.25% sodium-deoxycholate, 1 μM PMSF, 1 μM Na_3_VO_4_, 1 mM NaF, 0.7% Triton X-100, and 1 μg/ml of aprotinin, leupeptin, and pepstatin). Immunoprecipitation and Western blotting assays were performed as previously described [23]. Briefly, cell lysates were passed through a 26-gauge needle several times and detergent-insoluble materials were removed *via *centrifugation at 4^°^C and 12,000 x g. The protein concentration was then determined using a bicinchoninic acid assay (Pierce, Rockford, IL). Cell lysates were then immunoprecipitated with anti-p38 MAPK or anti-ERK 1/2 antibodies (Santa Cruz Biotechnology, Santa Cruz, CA) and subsequently resolved on an SDS-PAGE for analysis. The immunoprecipitate was then Western blotted with the anti-phosphotyrosine antibody (Upstate Biotechnology, Lake Placid, NY) to obtain a measure of the activation, as measured through phosphorylation, of p38 MAPK and ERK 1/2.

### Cell Stimulation, RNA Extraction and Reverse Transcription

CB NAMNC were incubated at 1x10^6^ NAMNC cells/ml in 100 x 20mm culture plates (Falcon Plastics, New Jersey, USA) at 37°C, 5% CO_2_ in the presence or absence of recombinant human IL-5 (1ng/ml) with or without 10ˉ^8^M BUD at varying time points. After stimulation, ice cold PBS was added, and cells were pelleted in a 15ml centrifuge tube (Sarstedt) at 1500rpm for 5 minutes at 4°C. The supernatant was poured off and RNA extracted using the Qiagen RNeasy mini-kit protocol for animal cells (Qiagen, Mississauga, Canada). Isolated RNA was treated with DNA-free (Ambion Inc., Canada), and the amount and purity determined using UV spectrophotometry (Ultrospec 2000, Fisher Scientific, Toronto, Canada). Two μg of total RNA from each condition was reverse transcribed using Oligo (dT) primers (StrataScript First-Strand Synthesis System, Stratagene, La Jolla, California, USA), and resulting cDNA diluted 1:5 with UltraPure distilled water (Gibco).

### Quantitative Real-Time PCR (Q-PCR)

Real-time PCR was carried out using the MX4000 machine (Stratagene), with SYBR Green chemistry (Stratagene) and run as a comparative quantitation experiment with GATA-1 primers (Search LC, Heidelberg, Germany). Positive and no-template controls, and the normalizing gene GAPDH (IDT, Iowa, USA) forward 5’- GAGTCAACG-GATTTGGTGT–3’, reverse GATTTTGG- AGGGACTCG 3’ were included with Human Reference Total RNA (Stratagene) used as a standard. Q-PCR reactions were set up as follows: 4ng cDNA was added to 12.5 μl 2x SYBR Green Master Mix, 100nM of forward and reverse primers and 30nM reference dye, giving a final volume of 25 μl. PCR cycles were 10 minutes at 95°C and 40 amplification cycles (95°C for 30 seconds and 55°C for 30 seconds). Target genes were confirmed using northern blot analysis, and the fold increase in GATA-1 gene expression between un-stimulated and stimulated cells was determined using the 2^–^∆∆Ct method [24].

### Statistics

All summary statistics are expressed as the mean ± SEM. For colony data, the mean colony counts are represented as the mean change from baseline (baseline was characterized as IL-5 alone). Group mean comparisons were performed on the absolute numbers using repeated measures analysis of variance (ANOVA). Statistical significance was assumed at a P value of less than 0.05. GATA-1 mRNA expression is reported as mean GATA-1 fold increases of ∆∆Ct of cells stimulated with IL-5 over un-stimulated cells. Comparisons between groups were made with ANOVA.

## RESULTS

### The Effects of BUD on IL-5-Responsive BM NAMNC

**Fig. (1):**
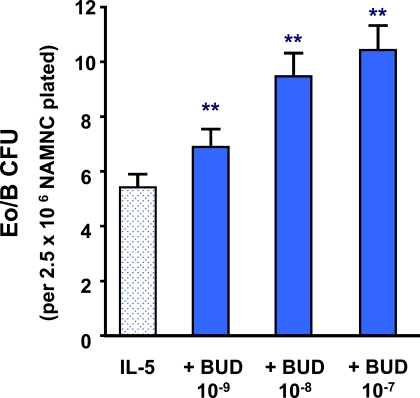
The effect of BUD on IL-5 stimulated bone marrow NAMNC. Error bars represent SEM (N= 34) (** p < 0.001).

The addition of BUD at concentrations of 10^-9^M to 10^-7^M caused a significant increase in IL-5-responsive Eo/B-CFU, compared to IL-5 alone (p<0.001) (Fig. **1**). In addition, preincubation of bone marrow cells with BUD (10^-9^M) *prior* to the addition of IL-5 also increased the number of Eo/B-CFU, compared to diluent control (p<0.0001) (data not shown).

### The Effects of BUD on IL-5- Responsive CB CD34^+^ Progenitors

**Fig. (2):**
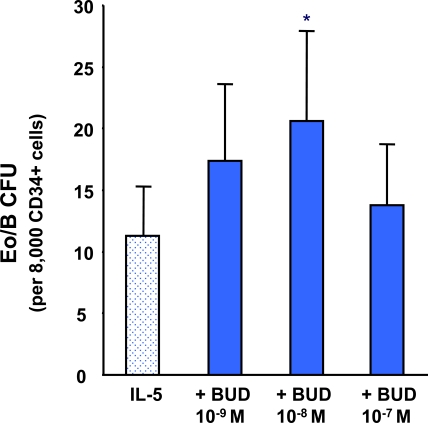
The effect of BUD on IL-5 stimulated CB-derived CD34+ progenitor cell differentiation. Error bars represent SEM (N=8) (*p < 0.001).

The addition of BUD, at concentrations of 10^-9^M to 10^-7^M caused an increase in IL-5-responsive Eo/B CFU compared to IL-5 alone reaching statistical significance at 10^-8^M (p<0.001) (Fig. **2**).

### The Effects of BUD on the IL-5Rα Intensity Expression on CB-Derived CD34^+^ Progenitor Cells and Bone Marrow NAMNC

The addition of BUD had no effect on the expression of IL-5Rα on CB-derived CD34^+ ^cells or bone marrow NAMNC, as measured by flow cytometry (data not shown).

### The Effects of BUD on IL-5-Transduced Activation of the Signaling Molecules p38 and ERK 1/2 Using Bone Marrow-Derived CD34^+^ Progenitor Cells

**Fig. (3):**
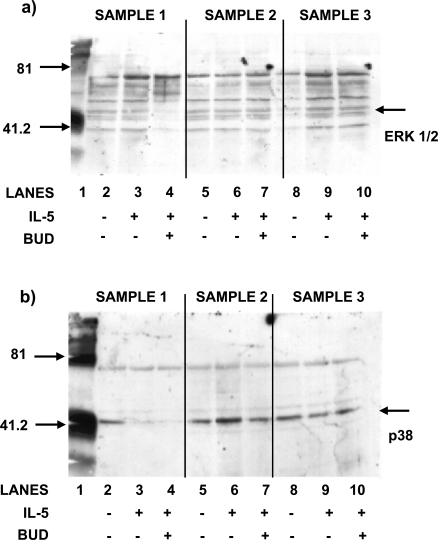
**(a)** The effect of BUD on the phosphorylation of ERK1/2. Bone marrow CD34^+^ cells were stimulated with IL-5 in the presence or absence of BUD (10^-8^M). Western blot using anti-ERK antibodies show that there is phosphorylation in sample three only (lane 9) and BUD has no effect (lane 10). **(b)** The effect of BUD on the phosphorylation of p38 MAPK. Bone marrow CD34^+^ cells were stimulated with IL-5 in the presence or absence of BUD (10^-8^M).Western blot using anti-p38 antibodies show that there is phosphorylation in sample two only (lane 6) and BUD has no effect (lane 7).

The addition of BUD at concentrations of 10^-8^M was shown to have no effect in either stimulating or inhibiting signal molecule activation, transduced *via *IL-5 interaction with IL-5Rα (N=3). Western blot analysis showed that phosphorylation of ERK 1/2 was detected in only one out of three samples stimulated with IL-5 at a concentration of 1ng/ml (lane 9, Fig. **3a**).

The addition of BUD, at a concentration of 10^-8^M, was shown to have no effect on the phosphorylation of ERK 1/2 in any of the three samples (lanes 4, 7 and 10; Fig. **3a**). Western blot analysis indicated that p38 MAPK was activated in only one of the three samples stimulated with IL-5 at a concentration of 1 ng/ml (lane 6, Fig. **3b**).

### The Effect of BUD on GATA-1 mRNA Expression

**Fig. (4):**
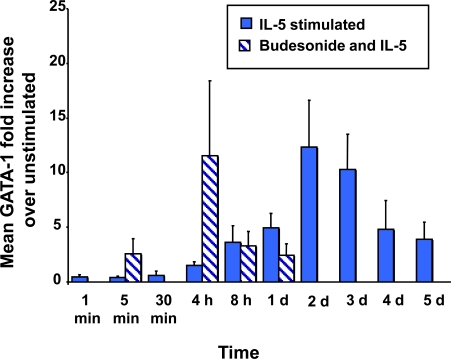
Effect of BUD on IL-5-stimulated GATA-1 expression. The graph summarizes the kinetics of GATA-1 expression in IL-5-stimulated CB hemopoietic progenitors. The effect of BUD on GATA-1 is shown in hatched bars.

IL-5 alone induces GATA-1 up-regulation in CB NAMNC, which peaks at 48 hours post-stimulation, with a 12.3 fold (±4.3) increase from baseline. The addition of BUD at a concentration of 10^-8^M resulted in an early enhancement of GATA-1 expression at 4 hours, with a fold increase of 11.5 (±6.8), followed by an inhibitory effect at 8 hours (3.2±1.3), returning to baseline levels at 24 hours (2.4±1.0). All results are in comparison to un-stimulated CB cells (Fig. **4**).

## DISCUSSION

Topical corticosteroids are currently the gold standard of therapy for the treatment of allergic diseases such as asthma and allergic rhinitis. They have potent effects *in vivo*, exerting their effects on a wide array of cell types, including Eo/B progenitors, which produce the predominant effector cell in allergic inflammation.

One of the aims of this study was to assess *in vitro* the direct effects of the glucocorticoid, BUD, on IL-5-mediated differentiation of bone marrow and CB-derived Eo/B progenitors. Our results show an *increase* in the number of lineage-committed, IL-5-responsive Eo/B progenitors, defined as an enhancement in Eo/B CFU, in the presence of BUD.

These results may appear counterintuitive, since inhaled steroid treatment has been shown to have a suppressive effect on circulating, bone marrow and tissue hemopoietic progenitors [9,12]. Inhibition of Eo/B progenitors has also been seen in tissue samples, since intranasal corticosteroid treatment results in increased CD34^+^/CD45^+^ cell immunostaining in nasal polyp sections; this is interpreted as evidence for arrested CD34^+^ differentiation, since there is accompanying reduction of mature eosinophil numbers in tissue [25]. In this context, it is important to note that airway eosinophils, while clearly markers of airway inflammation/exacerbation risk, may not always be directly involved in the pathogenesis of asthma, as evidenced by studies reporting persistent asthmatic responses in patients despite near ablation of blood and tissue eosinophils by anti-IL-5 therapy [26]; this could also be related to the incomplete effect of anti-IL-5 on eosinophil differentiation [27], or to confounding corticosteroid effects, or to a combination of these factors. In addition, Kuo *et al*. [28] have shown that in cultures of peripheral blood non-adherent, non-T-cell populations, dexamethasone at 10^-6^M causes a significant *decrease* in eosinophil CFU; however, these cultures were not supplemented with an exogenous colony stimulating factor, which likely accounts for the conflicting results.

Other* in vitro* studies have also given some conflicting data on the role of corticosteroids on eosinophils and their progenitors. For example, the addition of hydrocortisone (10^-5^M) to cultures of peripheral blood and bone marrow NAMNC, resulted in greater than 95% inhibition in eosinophil colony formation [29]. Slovick *et al*. demonstrated, using unfractionated bone marrow, an inhibition in the number of eosinophil colonies in cultures with hydrocortisone at 10^–6^M [15]. Of note in this latter study, hydrocortisone at the same concentration and in the presence of conditioned media (a source of colony stimulating activity), actually stimulated an increase in eosinophil colonies in bone marrow depleted of monocytes and T cells. Barr *et al*. [17] described the enhancing effects of physiological and pharmacological concentrations of hydrocortisone (10^-7^M and 10^-6^M) in increasing eosinophil colony numbers from cultures of human bone marrow. This was further substantiated by Butterfield *et al*. [30] who showed that incubation of bone marrow cells with micromolar concentrations of hydrocortisone resulted in a stimulation of eosinophil colony numbers. It was also shown in that study that administration of oral prednisone, while causing a significant decrease in bone marrow and peripheral blood eosinophil numbers, was not able to inhibit peripheral blood and bone marrow eosinophil colony numbers, thereby supporting the pro-eosinopoietic role of corticosteroids.

In a murine model, Gaspar Elsas *et al*. [18] demonstrated that the addition of dexamethasone (10^–7^M) to cultures of bone marrow from naïve mice caused a significant *increase* in GM-CSF-induced myeloid colony formation. Specifically, dexamethasone at 10^-8^M and 10^-7^M, resulted in a significant increase both in the frequency of pure eosinophil colonies and in the size of eosinophil colonies. In order to confirm these results, normal, naïve mice were administered dexamethasone *in vivo*, and bone marrow was harvested 24 hours later. Cultures of bone marrow cells yielded an increase in GM-CSF-induced colony formation, as well as in eosinophil colony size. Therefore, both *in vitro *and *in vivo* treatment with dexamethasone can result in selective enhancement of the eosinophil lineage and in myelopoiesis in general.

As a means of identifying a potential mechanism of the enhancing action of BUD on IL-5-mediated Eo/B differentiation, we focused on two mechanisms which increase IL-5 signaling through IL-5Rα on progenitor cells. The first, the expression of IL-5Rα on CD34+ progenitor cells, was examined since there is an increase in the proportion of bone marrow CD34^+^ progenitor cells expressing IL-5Rα 24 hours after allergen challenge in asthmatic subjects [10], and pretreatment with BUD *in vivo *partially reduces baseline levels of CD34^+^IL-5Rα^+^ bone marrow cells, but does not abrogate the allergen-induced upregulation [9]. Upham *et al*. demonstrated that the retinoic acid-induced reduction in the number of cells expressing IL-5Rα is accompanied by a reduction in the intensity of expression as measured by relative fluorescence intensity [31]. However, our current results indicate that BUD, at 10^-8^M, is unable to stimulate an increase in the intensity of expression of IL-5Rα on CB CD34^+^ progenitor cells as measured by sMFI. As a result, we conclude that BUD does not mediate its enhancing effects *via *increased IL-5Rα up-regulation or receptor density expression.

The second mechanism examined was the effect of BUD on IL-5 signaling *via *IL-5Rα. IL-5 signal transduction has been characterized previously by Adachi and Alam [32]. There are two main pathways of IL-5 signal transduction, the Ras-MAPK pathway and the JAK-STAT pathway [23,33]. IL-5 signaling *via *the Ras-MAPK pathway, specifically on the phosphorylation and activation of the ERK 1/2 and p38 signaling molecules, is critical for eosinophil function, including eosinophil survival, activation, degranulation and chemotaxis [23,34,35]. We hypothesized that BUD may mediate its enhancing effects *via *increased IL-5 signaling, and that this would lead to an increase in signaling molecules p38 and ERK 1/2 phosphorylation and activation. Our results demonstrated that in those samples where there was IL-5- transduced phosphorylation of p38 MAPK or ERK 1/2, the addition of BUD resulted in no measurable increases in phosphorylation by Western blotting. One possible problem is that we were unable to consistently attain baseline levels of IL-5-induced phosphorylation in our samples. However, there is no *a priori* evidence to suggest that BUD would cause an inhibition of p38 MAPK or ERK 1/2 phosphorylation, and this is supported by the fact that IL-5 signaling events are necessary for the observed BUD enhancing effects. With the numerous pathways and signaling molecules involved in the IL-5 signal transduction cascade, we are not able to definitively eliminate increased signaling through p38 MAPK or ERK 1/2 as a mechanism for our observed BUD-mediated enhancement of Eo/B CFU.

The kinetics of IL-5-induced GATA-1 expression have not been previously described. The promoter region of the IL-5 receptor contains binding sites for different transcription factors, Ap1, AP-1, GATA-1, and PU.1 [36]. GATA transcription factors are a family of factors with a conserved zinc-finger domain that bind DNA consensus GATA sequences [37]. GATA-1 is expressed in erythroid cells, megakaryocytes, mast cells and eosinophils [38] as well as Sertoli cells, and is a key transcription factor for the development of eosinophils from CD34^+^ hemopoietic progenitors. CD34^+ ^CB cells infected with an adenoviral vector containing GATA-1 were strongly supported into eosinophil differentiation. Moreover, the deletion of high-affinity GATA-binding sites within the GATA-1 promoter in a murine model leads to eosinophil loss [39]. Our results show that the peak of GATA-1 mRNA expression was at 2 days of IL-5 incubation. The addition of BUD led to a GATA-1 mRNA peak at a much earlier time point (4 hours); this may explain the increase in Eo/B CFU consistently seen with *in vitro* BUD whether CB or bone marrow progenitors are utilized. Similar glucocorticoid effects on transcription factor-induced cytokine expression have been described elsewhere [40]. In studies of stem-cell factor (SCF) regulation, the addition of BUD lead to a decrease in SCF mRNA expression while the addition of IL-1β resulted in an initial increase, which could be blocked by RU486, a glucocorticoid antagonist [40]. The authors speculated that potentiation by BUD of the IL-1β–induced SCF expression was related to an increased stability of SCF mRNA, and increased SCF gene transcription. A similar explanation may underlie BUD potentiation of IL-5-induced GATA-1 mRNA expression in our study. Moreover, since GATA-1 is auto-regulatory, in that it can positively regulate its own promoter [41,42], an earlier peak of GATA-1 expression and activity induced by BUD could be amplified and lead to increases in Eo/B differentiation.

## CONCLUSION

In summary, we have demonstrated that BUD leads to an increase in Eo/B lineage commitment. While the reasons for this may be multiple, we have shown that an initial increase in GATA-1 mRNA expression in IL-5-stimulated Eo/B progenitors may underlie the phenomenon. Further studies to examine Eo/B lineage-specific transcription factor expression and regulation in Eo/B progenitors may clarify the role of corticosteroids in Eo/B differentiation, and lead to more rational therapy for allergic diseases.
